# Oral Health and Discrimination Experienced by Sexual and Gender Minorities in Brazil: A Cross‐Sectional Study

**DOI:** 10.1111/scd.70133

**Published:** 2025-12-28

**Authors:** Igor Campos Guimarães, Ana Carla Batista Domiciano, Isabel Cristina Gonçalves Leite

**Affiliations:** ^1^ Master's Student At Collective Health Post‐Graduation Program Medicine School Department of Collective Health Federal University of Juiz de Fora Juiz de Fora Minas Gerais Brazil; ^2^ Dental Student At Dentistry school Federal University of Juiz de Fora Juiz de Fora Minas Gerais Brazil; ^3^ Medicine School Department of Collective Health Federal University of Juiz de Fora Juiz de Fora Minas Gerais Brazil

**Keywords:** indicators of quality of life, oral health, perceived discrimination, sexual and gender minorities

## Abstract

**Introduction:**

Sexual and gender minorities are victims of discrimination in health services, which might be associated with disparities in subjective oral health measures.

**Aims:**

This cross‐sectional study aimed to identify the association between different forms of discrimination experienced by Brazilian LGBTQIA+ people, and other independent variables, with subjective oral health outcomes.

**Methods and Results:**

Seven hundred sixty‐two individuals responded to an online questionnaire about their sociodemographic characteristics, discriminatory experiences on a daily basis and in dental services, access to these services, and oral health. Binary and multinomial logistic regression analyses estimated the association between independent variables and outcomes. The results were presented in odds ratios (OR) and respective 95% confidence intervals (95% C.I.). The emotional state was the category with the highest prevalence of impact (39.8%). Discrimination in dental services and self‐perceived need for treatment were associated with both self‐perceived oral health and its impacts on daily activities. Gender identity was associated with self‐perceived oral health, and sexual orientation was associated with oral impacts on daily performance.

**Conclusion:**

The study indicated oral health disparities in this population, associated with discrimination, gender identity, and sexual orientation, and the need for more welcoming dental services.

## 1 Introduction

The LGBTQIA+ population, composed of lesbian, gay, bisexual, asexual, transgender, and intersex people and other sexual and gender minorities [1, 2], suffers discrimination in different contexts [3, 4, 5, 6], including health services [7, 8, 9] and, specifically, dental services [10, 11, 12].

These events are conceptualized in the “minority stress model,” which distinguishes between distal stressors (such as discrimination) and proximal stressors (such as the expectation of discriminatory events), which might affect the health of minority groups [13]. It is also suggested that these stressors are more prevalent in young individuals from sexual minorities [14]. Discrimination and minority stress are associated with disparities in physical health [5, 6, 14, 15], including oral health [5, 6, 7, 16, 17].

Among the oral health evaluation measures are subjective instruments, such as self‐perceived oral health [10, 11, 18], and quality of life instruments related to oral health, such as the Oral Health Impact Profile (OHIP‐14) [10] and the Oral Impacts on Daily Performance (OIDP) [16, 19].

Compared to heterosexual cisgender individuals, sexual minorities have a higher prevalence of fair or poor self‐perceived oral health [18], and 46% of a Brazilian LGBTQIA+ people sample reported some level of satisfaction with their self‐perceived oral health [10]. Research also points to a synergistic interaction between psychological stress, discrimination, and oral health impacts on daily activities [16].

A study suggests that, even in the general population, the lack of access to dental services is associated with worse outcomes in these measures, pointing to a worse quality of life related to oral health [20]. This is a common finding in relation to LGBTQIA+ people, since individuals with difficulty in accessing services had twice the chance of having this parameter impacted [10].

Therefore, the objective of the present research was to identify the association between the discrimination experienced by LGBTQIA+ people in daily life and in dental services with subjective oral health outcomes.

## 2 Materials and Methods

This was a cross‐sectional study, conducted in Brazil between November 2024 and May 2025, after the approval of the Research Ethics Committee of the authors’ university. The article was written following the recommendations of the “STROBE Declaration” [21].

Data were collected via an online questionnaire on the Google Forms platform (Mountain View, California, United States), which was shared by 92 LGBTQIA+ associations, organizations, groups, social centers, cultural and advocacy events' social media from capitals and countryside cities across every Brazilian state, aiming to reach a large number of volunteers and enable a convenience sample with the maximum representation of Brazilian states.

These institutions shared the research questionnaire link through their social media using standardized recruitment images provided by the authors. These images contained an attention‐grabbing phrase (for example, “Do you self‐identify as an LGBTQIA+ person? Could you help us to gain an overview about dental services access and oral health in Brazil?”), basic information about the survey, a QR Code, and a link to the questionnaire. Dissemination could also occur in their physical spaces through flyers or informative posters, whenever possible.

Prior to accessing the questionnaire, participants read and agreed to the Free and Informed Consent Form. In case of disagreement, candidates were informed that they could close the form without any harm and/or negative consequences.

The questionnaire, with 59 questions and divided into four parts, contained questions regarding sociodemographic characterization [22, 23]; discrimination in health services, adapted from questions from the National Health Survey of 2013 [24, 25] and the research “Mapping of Trans People in the City of São Paulo” (*Mapeamento de Pessoas Trans na Cidade de São Paulo*) [26], as well as the discrimination experienced in daily baisis, using the Everyday Discrimination Scale (EDS) [27]; and questions from the National Oral Health Survey [23] regarding access to dental services and self‐perceived oral health and its impacts on daily activities (OIDP). Each question had a “don't know/prefer not to answer” response option.

The variables created from these questions were hierarchically organized into six blocks, based on the Minority Stress Model [13]. These blocks were sequentially included in statistical analyses ranging from distal to more proximal stressors, as they gradually interact with each other. Block 1 controlled the associations by sociodemographic confounding factors. The variables “biological sex” and “gender identity” were used only as descriptive variables. For the bivariate and multivariate analyses, the “gender” variable was chosen, dichotomized into “cisgender” and “transgender, non‐binary or other gender identity”. Block 2 gathered the variables “education” and “monthly family income” to control for economic and education factors. Block 3 contained the Everyday Discrimination Scale, which was categorized by its quartile distribution, and, in the fourth, the experience of discrimination in dental services. Block 5 defined how the dental service access occurred. Block 6 categorized whether respondents sought these services, had difficulty accessing them, and used them recently.

The OIDP instrument was dichotomized, according to a unidimensional analysis previously validated in Brazil [28], into “with impact” (defined by a positive response in at least one dimension) and “without impact” (defined by its absolute absence). Self‐perceived oral health was grouped into positive, for the responses “very good” and “good,” negative, in reference to “poor” and “very poor,” and fair.

A minimum satisfactory sample of 455 people was estimated based on the estimates of LGBTQIA+ people in Brazil [1, 29, 30], the lowest prevalence of discrimination in the analyzed studies [3, 9, 26] and the adoption of a significance level of 5% (α = 0.05) and a test power of 80% (β = 0.2). The inclusion criteria were individuals with an age greater than 18 years who indicated the sexual orientation, gender identity, and/or biological sex consistent with the LGBTQIA+ population. The exclusion criteria were participants who indicated the “heterosexual” option and did not indicate the “transgender,” “non‐binary”, or “intersex” options.

The data were organized and analyzed in the statistical program Statistical Package for the Social Sciences (SPSS), 20.0 for Windows (SPSS Inc., Chicago, IL, USA). The descriptive analysis was performed by establishing the absolute and relative frequencies for categorical variables and the minimum and maximum values, mean, quartiles, and standard deviation for numerical variables. The Kolmogorov–Smirnov, *U* of Mann–Whitney, Chi‐square, *t*‐test for group comparisons, and binary and multinomial logistic regression analyses were used for the statistical analysis of the associations between the dependent and independent variables, adopting 95% confidence intervals (95% C.I.) and a significance level of 5% (*p* < 0.05). The binary logistic regression analysis was performed for the OIDP outcome, and the multinomial logistic regression analysis was performed for the self‐perceived oral health.

Only the variables that maintained a statistically significant association in the previous stage were included in the hierarchical multivariate and final models. Variables that lost association in each model were not reported in the corresponding level column of the second and third tables.

## 3 Results

The research form obtained 787 responses, reduced to 762 after the application of inclusion and exclusion criteria. Ten cisgender heterosexual participants who were not intersex, transgender or non‐binary and 15 who completed a birth date incompatible with the minimum age of the research were not included in the final sample.

Table 1 presents the distribution of the independent variables in relation to the outcomes, and Tables 2 and 3 present the crude and adjusted association measures for the outcomes.

**TABLE 1:** Descriptive statistics distributed according to subjective oral health outcomes.

	OIDP, *n* = 758^a^			Self‐perceived oral health, *n* = 761^a^			
Variables	Without impact, *n* = 301^b^	With impact, *n* = 457^b^	*p* ^c^	Positive, *n* = 421^b^	Fair, *n* = 248^b^	Negative, *n* = 92^b^	*p* ^c^
**Block 1**							
Age group			0.469				0.177
18 to 24 years old	69 (22.92)	126 (27.6)		105 (24.9)	66 (26.6)	25 (27.2)	
25 to 39 years old	185 (61.46)	261 (57.1)		261 (62)	141 (56.9)	45 (48.9)	
40 to 59 years old	45 (14.95)	65 (14.2)		52 (12.4)	38 (15.3)	51 (22.8)	
60 years or older	2 (0.67)	5 (1.1)		3 (0.7)	3 (1.2)	1 (1.1)	
Color/race^a^			0.379				0.213
Yellow	4 (1.3)	7 (1.55)		9 (2.15)	2 (0.8)	0 (0)	
Indigenous	5 (1.7)	10 (2.21)		9 (2.15)	6 (2.4)	0 (0)	
Black/brown	124 (41.6)	214 (47.34)		176 (42)	118 (47.8)	45 (51.7)	
White	165 (55.4)	221 (48.9)		225 (53.7)	121 (49)	42 (48.3)	
Biological sex^a^			0.181				0.141
Male	178 (59.7)	242 (54.14)		247 (59.2)	125 (51.9)	51 (56.7)	
Female	120 (40.3)	203 (45.41)		170 (40.8)	115 (47.7)	38 (42.2)	
Intersex	0 (0)	2 (0.45)		0 (0)	1 (0.4)	1 (1.1)	
Gender^a^			< 0.001				< 0.001
Transgender / non‐binary / other	51 (17.5)	149 (32.9)		64 (15.5)	85 (34.8)	51 (56)	
Cisgender	241 (82.5)	304 (67.1)		349 (84.5)	159 (65.2)	40 (44)	
Gender identity^a^			< 0.001				< 0.001
Cisgender man	151 (52.25)	184 (41.5)		219 (53.5)	93 (39.24)	26 (29.21)	
Cisgender woman	90 (31.14)	120 (27.1)		130 (31.8)	66 (27.85)	14 (15.73)	
Transgender man	13 (4.5)	47 (10.6)		216 (3.9)	31 (13.08)	13 (14.61)	
Transgender woman	15 (5.19)	35 (7.9)		15 (3.7)	19 (8.02)	16 (17.98)	
Transgender / non‐binary / other	20 (6.92)	55 (12.4)		29 (7.1)	27 (11.39)	19 (21.35)	
Transgender Intersex	0 (0)	2 (0.5)		0 (0)	1 (0.42)	1 (1.12)	
Sexual orientation^a^			< 0.001				< 0.001
Heterosexual	16 (5.35)	42 (9.2)		18 (4.3)	23 (9.3)	17 (18.5)	
Bisexual / asexual / other	81 (27.09)	187 (41)		116 (27.7)	106 (42.9)	46 (50)	
Homosexual	202 (67.56)	227 (49.8)		285 (68)	118 (47.8)	29 (31.5)	
Region of residence^a^			0.001				0.003
North	19 (6.31)	65 (14.3)		37 (8.8)	33 (13.3)	14 (15.2)	
Northeast	63 (20.93)	121 (26.5)		47 (20.7)	69 (27.8)	29 (31.5)	
Central‐west	35 (11.63)	57 (10.3)		87 (11.2)	26 (10.5)	10 (10.9)	
South	54 (17.94)	72 (15.8)		67 (16)	49 (19.8)	11 (12)	
Southeast	130 (43.19)	151 (33.1)		142 (43.3)	71 (28.6)	28 (30.4)	
**Block 2**							
Household income^a^			< 0.001				<0.001
Up to 2400 reais	57 (19.4)	153 (34.7)		73 (17.4)	89 (37.6)	48 (55.2)	
Between 2401 and 4000 reais	67 (22.8)	106 (24)		100 (24.2)	52 (21.9)	22 (25.3)	
Between 4001 and 7000 reais	73 (24.8)	92 (20.9)		110 (26.6)	46 (19.4)	9 (10.3)	
Above 7001 reais	97 (33)	90 (20.4)		130 (31.5)	50 (21.1)	8 (9.2)	
Education^a^			< 0.001				< 0.001
Up to complete elementary school	0 (0)	13 (2.9)		6 (1.4)	4 (1.6)	3 (3.26)	
Up to complete high school	35 (11.6)	79 (17.3)		42 (10)	49 (19.8)	23 (25)	
Incomplete higher education	70 (23.3)	140 (30.7)		103 (24.5)	74 (30)	33 (35.87)	
Complete higher education	196 (65.1)	224 (49.1)		270 (64.1)	120 (48.6)	33 (35.87)	
**Block 3**							
Everyday discrimination scale			< 0.001^c^				< 0.001^c^
First quartile	103 (34.2)	79 (17.3)		135 (32.1)	34 (13.7)	13 (14.1)	
Second quartile	90 (29.9)	102 (22.3)		109 (25.9)	65 (26.2)	19 (20.7)	
Third quartile	70 (23.3)	115 (25.2)		101 (24)	63 (25.4)	22 (23.9)	
Fourth quartile	38 (12.6)	161 (35.2)		76 (18.1)	86 (34.7)	38 (41.3)	
**Block 4**							
Discrimination in dental services^a^			< 0.001				< 0.001
Suffered	55 (18.4)	234 (51.9)		111 (26.6)	112 (45.9)	67 (73.6)	
Did not suffer	244 (81.6)	217 (48.1)		307 (73.4)	132 (54.1)	24 (26.4)	
**Block 5**							
Hesitation in seeking dental services^a^			< 0.001				< 0.001
Yes	21 (7.1)	107 (24.5)		32 (7.8)	57 (24.2)	39 (43.8)	
No	275 (92.9)	329 (75.5)		378 (92.2)	179 (75.8)	50 (56.2)	
Type of dental service sought^a^			0.008				< 0.001
Public service	37 (17.1)	83 (26.9)		50 (15.2)	46 (30.3)	24 (51.1)	
Private service/health plan/other	180 (82.9)	225 (73.1)		278 (84.8)	106 (69.7)	23 (48.9)	
Main reason for last appointment^a^			< 0.001				< 0.001
Toothache	3 (1)	40 (8.97)		12 (2.9)	20 (8.4)	12 (13.19)	
Exodontia	13 (4.4)	53 (11.88)		20 (4.8)	32 (13.44)	14 (15.38)	
Treatment	63 (21.1)	169 (37.9)		113 (27)	74 (31.1)	45 (49.45)	
Others	9 (3)	20 (4.48)		13 (3.1)	11 (4.62)	5 (5.5)	
Check‐up and/or prevention	210 (70.5)	164 (36.77)		260 (62.2)	101 (42.44)	15 (16.48)	
Last dental appointment evaluation^a^			< 0.001				< 0.001
Negative	7 (2.35)	33 (7.4)		6 (1.44)	15 (6.3)	19 (21.1)	
Fair	26 (8.72)	110 (24.8)		46 (11.03)	60 (25.2)	30 (33.3)	
Positive	265 (88.93)	301 (67.8)		365 (87.53)	163 (68.5)	41 (45.6)	
Self‐perceived dental treatment need^a^			< 0.001				< 0.001
Yes	181 (61.4)	417 (92.7)		283 (68.4)	255 (93)	91 (100)	
No	114 (38.6)	33 (7.3)		131 (31.6)	17 (7)	0 (0)	
Self‐perceived dental treatment need reason^a^			< 0.001				< 0.001
Check‐up and/or prevention	149 (66.8)	156 (36.1)		195 (58.73)	96 (41.8)	15 (16.3)	
Toothache	1 (0.4)	10 (2.3)		5 (1.51)	5 (2.15)	1 (1.1)	
Exodontia	8 (3.6)	33 (7.6)		21 (6.33)	16 (6.9)	4 (4.35)	
Treatment	61 (27.4)	218 (50.5)		104 (31.32)	108 (46.55)	67 (72.82)	
Others	4 (1.8)	15 (3.5)		7 (2.11)	7 (3.02)	5 (5.43)	
**Block 6**							
Seeking dental services in the last year^a^			0.155				< 0.001
Did not seek	82 (27.4)	146 (32.3)		89 (21.3)	96 (38.9)	44 (48.9)	
Sought	217 (72.6)	306 (67.7)		328 (78.7)	151 (61.1)	46 (51.1)	
Difficulty in accessing dental services^a^			< 0.001				< 0.001
Yes	4 (1.8)	37 (12.1)		12 (3.7)	13 (8.6)	16 (34.8)	
No	213 (98.2)	269 (87.9)		316 (96.3)	138 (91.4)	30 (65.2)	
Recent use of dental services^a^			0.043				< 0.001
More than 1 year	86 (29.2)	163 (36.3)		90 (21.7)	103 (42.7)	56 (61.5)	
Up to 1 year	209 (70.8)	286 (63.7)		325 (78.3)	138 (57.3)	35 (38.5)	

^a^ Missing cases excluded.

^b^ *n* (%).

^c^ Bivariate association *p* value.

**TABLE 2:** Crude and adjusted association measures for self‐perceived oral health.

	Fair self‐perceived oral health^b^						Negative self‐perceived oral health^b^					
Variables	Crude OR (95% C.I.)^a^	*p* ^c^	Adjusted OR in the block (95% C.I.)^a^	*p* ^c^	Final model adjusted OR (95% C.I.)^a^	*p* ^c^	Crude OR (95% C.I.)^a^	*p* ^c^	Adjusted OR in the block (95% C.I.)^a^	*p* ^c^	Final model adjusted OR (95% C.I.)^a^	*p* ^c^
**Block 1**												
Gender		< 0.001		< 0.001				< 0.001		< 0.001		0.016
Transgender / non‐binary / other	2.91 (2.00–4.24)		2.24 (1.41–3.56)				6.95 (4.25–11.38)		4.54 (2.48–8.41)		4.25 (1.31–13.76)	
Cisgender	1		1				1		1		1	
Sexual orientation												
Heterosexual	3.09 (1.61–5.93)	< 0.001	1.44 (0.66–3.15)	0.364	1.05 (0.40–2.76)	0.912	9.28 (4.32–19.95)	< 0.001	2.26 (0.90–5.70)	0.084		
Bisexual / asexual / other	2.21 (1.57–3.10)	< 0.001	1.83 (1.26–2.66)	0.001	1.57 (1.01–2.44)	0.045	3.90 (2.33–6.51)	< 0.001	2.32 (1.31–4.12)	0.004		
Homosexual	1		1		1		1		1			
Region of residence												
North	2.29 (1.33–3.94)	0.003	2.15 (1.23–3.75)	0.007	2.01 (1.04–3.89)	0.039	2.46 (1.18–5.12)	0.016	2.06 (0.94–4.51)	0.072	7.27 (1.62–32.66)	0.010
Northeast	2.03 (1.34–3.09)	< 0.001	1.98 (1.28–3.06)	0.002	1.85 (1.09–3.14)	0.022	2.17 (1.21–3.86)	0.009	1.96 (1.06–3.62)	0.032	6.09 (1.48–25.01)	0.012
Central‐west	1.42 (0.82–2.46)	0.215	1.27 (0.71–2.26)	0.421	1.33 (0.67–2.64)	0.409	1.38 (0.63–3.05)	0.421	1.12 (0.48–2.59)	0.790	3.92 (0.70–22.04)	0.120
South	1.87 (1.18–2.97)	0.007	1.86 (1.15–3.00)	0.012	2.05 (1.16–3.64)	0.014	1.07 (0.50–2.26)	0.865	1.06 (0.485–2.33)	0.879	2.83 (0.53–14.92)	0.221
Southeast	1		1		1		1		1		1	
**Block 2**												
Household income												
Up to 2400 reais	3.17 (2.02–4.97)	< 0.001	2.65 (1.62–4.31)	< 0.001	2.08 (1.14–3.79)	0.016	10.68 (4.79–23.81)	< 0.001	7.82 (3.35–18.24)	< 0.001		
Between 2401 and 4000 reais	1.35 (0.85–2.16)	0.206	1.19 (0.74–1.93)	0.472	0.82 (0.45–1.47)	0.504	3.57 (1.53–8.37)	0.003	3.01 (1.27–7.16)	0.012		
Between 4001 and 7000 reais	1.09 (0.68–1.75)	0.729	1.03 (0.64–1.66)	0.910	0.84 (0.48–1.48)	0.381	1.33 (0.50–3.56)	0.571	1.20 (0.45–3.24)	0.716		
Above 7001 reais	1		1		1		1		1			
Education												
Up to complete elementary school	1.50 (0.42–5.41)	0.536	0.61 (0.14–2.59)	0.504			4.09 (0.98–17.13)	0.054	1.43 (0.32–6.41)	0.637		
Up to complete high school	2.62 (1.65–4.18)	< 0.001	1.79 (1.07‐3.00)	0.027			4.48 (2.40–8.36)	< 0.001	2.18 (1.09–4.36)	0.028		
Incomplete higher education	1.62 (1.20–2.34)	0.011	1.34 (0.91–1.98)	0.140			2.62 (1.54–4.47)	< 0.001	1.70 (0.95–3.04)	0.071		
Complete higher education	1		1				1		1			
**Block 3**												
Everyday discrimination scale												
First quartile	1		1		1		1		1		
Second quartile	2.37 (1.46–3.85)	< 0.001	2.37 (1.46–3.85)	< 0.001	2.57 (1.44–4.60)	0.001	1.81 (0.86–3.83)	0.121	1.81 (0.86–3.83)	0.121		
Third quartile	2.48 (1.51–4.04)	< 0.001	2.48 (1.51–4.04)	< 0.001	1.68 (0.91–3.09)	0.094	2.26 (1.09–4.71)	0.29	2.26 (1.09–4.71)	0.29		
Fourth quartile	4.49 (2.76–7.31)	< 0.001	4.49 (2.76–7.31)	< 0.001	2.04 (1.05–3.95)	0.035	5.19 (2.60–10.35)	< 0.001	5.19 (2.60–10.35)	<0.001		
**Block 4**												
Discrimination in dental services		< 0.001		<0.001				< 0.001		< 0.001		0.006
Suffered	2.35 (1.68–3.27)		2.35 (1.68–3.27)				7.72 (4.62–12.91)		7.72 (4.62–12.91)		4.76(1.57–14.48)	
Did not suffer	1		1				1		1		1	
**Block 5**												
Hesitation in seeking dental services		< 0.001		0.013				< 0.001		<0.001		
Yes	3.76 (2.36–6.01)		2.45 (1.24–4.88)				9.21 (5.30–16.01)		4.95 (2.08–11.80)			
No	1		1				1		1			
Type of dental service sought		< 0.001						< 0.001		0.004		
Public service	2.41 (1.52–3.82)						5.80 (3.04–11.07)		3.16 (1.44–6.96)			
Private service/health plan/other	1						1		1			
Main reason for last appointment												
Toothache	4.29 (2.02–9.10)	< 0.001	2.17 (0.79–5.96)	0.134			17.33 (6.67–45.02)	< 0.001	8.53 (1.70–42.86)	0.009		
Exodontia	4.12 (2.25–7.54)	< 0.001	2.50 (1.08–5.82)	0.033			12.13 (5.14–28.63)	< 0.001	13.31 (3.49–50.79)	<0.001		
Treatment	1.69 (1.16–2.45)	0.006	1.35 (0.82–2.20)	0.235			6.90 (3.70–12.89)	< 0.001	8.76 (3.09–24.80)	<0.001		
Others	2.18 (0.94–5.02)	0.068	1.36 (0.43–4.33)	0.605			6.67 (2.10–21.17)	< 0.001	1.60 (0.14–17.67)	0.703		
Check‐up and/or prevention	1		1				1		1			
Last dental appointment evaluation												
Negative	5.60 (2.13–14.69)	< 0.001	6.29 (1.57–25.20)	0.009	4.08(1.30–12.86)	0.016	28.19 (10.65–74.59)	< 0.001	15.66 (3.12–78.55)	< 0.001	9.98(1.33–74.92)	0.025
Fair	2.92 (1.91–4.47)	< 0.001	2.15 (1.19–3.86)	0.011	1.52 (0.90–2.57)	0.116	5.81 (3.31–10.18)	< 0.001	3.79 (1.62–8.88)	0.002	3.32 (1.12–9.90)	0.031
Positive	1		1		1		1		1		1	
Self‐perceived dental treatment need		< 0.001		<0.001		<0.001						
Yes	6.13 (3.59–10.46)		4.21 (2.16–8.19)		4.43 (2.36–8.33)							
No	1		1		1							
**Block 6**												
Seeking dental services in the last year		< 0.001						< 0.001				
Did not seek	2.34 (1.66–3.31)						3.52 (2.19–5.67)					
Sought	1						1					
Difficulty in accessing dental services		0.028						< 0.001		< 0.001		
Yes	2.48 (1.10–5.57)						14.04 (6.08–32.43)		7.04 (2.59–19.15)			
No	1						1		1			
Recent use of dental services		< 0.001						< 0.001		0.009		0.012
More than 1 year	2.69 (1.91–3.81)						5.78 (3.57–9.36)		4.05 (1.41–11.57)		7.39 (1.56–34.90)	
Up to 1 year	1						1		1		1	

^a^ O.R., odds ratio; 95% C.I., confidence interval.

^b^ Compared to positive self‐perceived oral health.

^c^ Multinomial logistic regression.

**TABLE 3:** Crude and adjusted association measures for oral health impacts on daily activities.

	Oral impacts on daily performances					
Variables	Crude OR (95% C.I.)^a^	*p* ^b^	Adjusted OR in the block (95% C.I.)^a^	*p* ^c^	Final model adjusted OR (95% C.I.)^a^	*p* ^c^
**Block 1**						
Gender		< 0.001		0.004		
Transgender / non‐binary / other	2.32 (1.62‐3.32)		1.95 (1.24‐3.08)			
Cisgender	1		1			
Sexual orientation						
Bisexual / asexual / other	2.05 (1.49‐2.84)	<0.001	1.64 (1.15‐2.34)	0.006	1.76 (1.04‐2.98)	0.036
Heterosexual	2.34 (1.27‐4.28)	0.006	1.18 (0.56‐2.50)	0.655	0.95 (0.28‐3.19)	0.932
Homosexual	1		1		1	
Region of residence						
North	2.94 (1.68‐5.17)	<0.001	2.68 (1.51‐4.75)	< 0.001		
Northeast	1.65 (1.13‐2.43)	0.010	1.62 (1.09‐2.41)	0.018		
Central‐west	1.16 (0.70‐1.90)	0.567	1.04 (0.62‐1.73)	0.887		
South	1.15 (0.75–1.75)	0.523	1.13 (0.73‐1.76)	0.577		
Southeast	1		1			
**Block 2**						
Household income						
Up to 2400 reais	2.89 (1.90‐4.39)	<0.001	2.17 (1.38‐3.41)	<0.001		
Between 2401 and 4000 reais	1.70 (1.21‐2.59)	0.013	1.49 (0.97‐2.29)	0.068		
Between 4001 and 7000 reais	1.36 (0.89‐2.07)	0.153	1.26 (0.82‐1.93)	0.289		
Above 7001 reais	1		1			
Education						
Up to complete elementary school	1413 (000‐)	0.998	9538 (000‐)	0.999		
Up to complete high school	1.97 (1.27‐3.07)	0.003	1.65 (1.01‐2.70)	0.047		
Incomplete higher education	1.75 (1.24‐2.47)	0.001	1.51 (1.05‐2.17)	0.026		
Complete higher education	1		1			
**Block 3**						
Everyday discrimination scale						
First quartile	1		1		1	
Second quartile	1.48 (0.98‐2.22)	0.061	1.48 (0.98‐2.22)	0.061	1.19 (0.65‐2.19)	0.566
Third quartile	2.14 (1.41‐3.15)	< 0.001	2.14 (1.41‐3.15)	< 0.001	2.08 (1.10‐3.94)	0.024
Fourth quartile	5.52 (3.49‐8.74)	< 0.001	5.52 (3.49‐8.74)	< 0.001	1.83 (0.86‐3.88)	0.116
**Block 4**						
Discrimination in dental services		< 0.001		< 0.001		0.031
Suffered	4.78 (3.38‐6.76)		4.78 (3.38‐6.76)		1.82 (1.05‐3.14)	
Did not suffer	1		1		1	
**Block 5**						
Hesitation in seeking dental services		< 0.001		0.013		
Yes	4.26 (2.60‐6.98)		2.62 (1.22‐5.64)			
No	1		1			
Type of dental service sought		0.008				
Public service	1.79 (1.16‐2.77)					
Private service/health plan/other	1					
Main reason for last appointment						
Toothache	17.07 (5.19‐56.17)	< 0.001	18.48 (2.39‐142.79)	0.005	18.66 (2.35‐147.89)	0.006
Exodontia	5.22 (2.75‐9.90)	< 0.001	4.39 (1.74‐11.08)	0.002	3.10 (1.18‐8.11)	0.021
Treatment	3.43 (2.41‐4.89)	< 0.001	2.70 (1.70‐4.29)	<0.001	2.24 (1.34‐3.73)	0.002
Others	2.84 (1.26‐6.41)	0.012	1.97 (0.66‐5.91)	0.226	1.52 (0.48‐2.92)	0.487
Check‐up and/or prevention	1		1		1	
Last dental appointment evaluation						
Negative	4.15 (1.80‐9.54)	< 0.001				
Fair	3.72 (2.36‐5.89)	< 0.001				
Positive	1					
Self‐perceived dental treatment need		< 0.001		< 0.001		<0.001
Yes	7.96 (5.20‐12.17)		5.00 (2.98‐8.38)		4.31 (2.49‐7.49)	
No	1		1		1	
**Block 6**						
Difficulty in accessing dental services		< 0.001		<0.001		0.039
Yes	7.32 (2.57‐20.87)		8.40 (2.62‐26.90)		4.01 (1.07‐15.00)	
No	1		1		1	
Recent use of dental services		0.044				
More than 1 year	1.38 (1.01‐1.90)					
Up to 1 year	1					

^a^ O.R., odds ratio; 95% C.I., confidence interval.

^b^ Pearson's Chi‐squared test.

^c^ Binary logistic regression.

The sample was primarily composed of cisgender people, homosexuals, with an age group between 25 and 39 years, with white skin color, residents of the Southeast region of Brazil, who have a monthly family income of up to 2400 reais, and with higher education. The Everyday Discrimination Scale (EDS) did not follow a parametric distribution, and most respondents fell into the fourth quartile (26.2%), followed by the second quartile (25.3%). The majority of the respondents who reported impacts on daily performance and a negative self‐perception of oral health were also situated in the fourth quartile of the EDS.

Regarding access to dental services, the majority did not suffer discrimination, did not hesitate to attend them due to not feeling accepted, did not seek public services, presented preventive or re‐evaluation reasons for the last appointment, evaluated this appointment positively, sought them and used them in the last year, and did not face difficulty in accessing them in the last year.

The majority presented a positive self‐perception of oral health (55.3%; 95% C.I. 52%–59%); however, a majority reported some impact of oral health on daily activities (60.3%; 95% C.I. 57%–64%). Forty‐four percent (95% C.I. 39%–49%) of the individuals with a positive self‐perception presented at least one daily activity impacted by oral health.

Seventeen percent reported only one daily activity impacted by oral health (17.4%; 95% C.I. 15%–20%), 15% (95% C.I. 13%–18%) presented five or more daily activities impacted by oral health. Emotional state (39.8%; 95% C.I. 36%–43%), teeth cleaning (33.9%; 95% C.I. 31%–37%), and confidence to smile or speak (31.2%; 95% C.I. 28%–34%) were the most impacted dimensions.

Positive self‐perceived oral health was the reference category for the analyses of this instrument. Transgender people, residents of the North and Northeast regions, who suffered discrimination in dental services, who evaluated the last appointment negatively and fairly, and who used these services for more than a year, presented higher odds of presenting a negative self‐perception of oral health.

People who reported their sexual orientation as bisexual, asexual or other; who were in the third quartile of the EDS; who suffered discrimination in dental services; for whom the main reason for the last appointment was toothache, exodontia or treatment; who report the need for treatment; and who had difficulty in accessing dental services presented higher odds of having impacts on daily activities resulting from oral health.

## 4 Discussion

The present research aimed to contribute to the understanding of the factors associated with self‐perceived oral health and the quality of life related to oral health of LGBTQIA+ people, an incipient field in Brazil [5, 10, 19], and other countries [6, 31, 32], by including variables related to access to services and the discrimination experienced in them and in daily life.

However, it presents limitations, such as the cross‐sectional design, which hinders the establishment of causality, and the sub‐representation of Black, Brown, Indigenous, Asian and Asian‐descent people, those who are transgender, non‐binary and other gender identities, those who live in Brazilian north and central‐west regions, those who are over 39 years of age, those who receive a low income and have lower levels of education. Further investigations might focus on those groups to better understand their experiences.

The survey propagation via LGBTQIA+ advocacy institutions' social media might have resulted in selection and volunteer bias of people who are most engaged with this topic, more digitally literate, and who are cisgender and have an advanced educational degree (which represents 55.5% of the sample). These characteristics might have led to an underestimation of the true prevalence of discrimination and poor oral health in the Brazilian LGBTQIA+ population.

Due to the transgender people subrepresentation, it was necessary to aggregate their subgroups (transgender men and women, non‐binary, and other gender identities), which could have caused a loss of information and nuance.

In addition, the sensitivity caused by discriminatory events, the fear of their recurrence, and discomfort upon recalling and reporting these experiences, as well as the self‐reported oral health outcomes, may have contributed to information bias.

In the comparison between individuals from sexual minorities and cisgender heterosexuals, the disparities in physical health could be partially explained by the occurrence of stressors based on the discrimination, threat, and violence that the minority group suffered [14].

Studies previously conducted with different population groups pointed to the association between discrimination and oral health [5, 6, 7, 16, 17], and resilience presented itself as a mediating and protective factor in this relationship [17].

In this research, the discrimination experienced in daily life by LGBTQIA+ people maintained an association in the final model of the logistic regression with fair self‐perceived oral health and the oral impacts on daily performance, which is an association that was also observed in a Brazilian study with beneficiaries of social programs [16].

Moreover, discrimination in dental services was associated with a 4.76 times greater chance of belonging to the negative self‐perceived oral health group and an 82% greater chance of suffering an impact on daily activities from oral health, which suggests that the negative experiences in dental services are a more proximal and direct stressor for oral health outcomes. The OIDP data were similar to those of a study with secondary health data from elderly Brazilians, which suggested an 85% higher risk of impact on daily activities in individuals discriminated against in health services [7].

Two studies conducted with Brazilian and Australian adolescents indicated, respectively, prevalences 16% [5] and 38% [6] for higher impact on oral health‐related quality of life in victims of discrimination due to sexual orientation.

Factors such as using dental services for more than a year and the evaluation of the last dental appointment, which presented an association with self‐perceived oral health in this research, might indicate a relationship between unsatisfactory experiences in dental services, their late use, and precarious oral health conditions [10, 33].

Among the sexual and gender minorities, transgender people presented worse oral health outcomes [10, 11, 19, 32, 34, 35]. Transgender people presented, in this research, 4.25 times more chances of having a negative self‐perception of oral health, a difference that was also similarly observed in other studies with the Brazilian LGBTQIA+ population, both with the same instrument [11, 19] and with oral health‐related quality of life instruments [10, 19].

Clinical dental disparities between the transgender and cisgender populations can support the subjective differences [32, 34]. In studies conducted in India, only 10% of transgender people presented a positive self‐perception of oral health [32]; 27.78% did not have carious teeth and 7.78% presented a healthy periodontium [35]; the carious component was the most prevalent in the CPO‐D index [32] and this group presented more oral lesions and a higher average in the CPO‐D index than cisgender people [34]. In Brazil, transgender people reported tooth loss more frequently than cisgender people from sexual minorities [11].

In the present research, the domains of the OIDP instrument that presented a higher prevalence of impact were the emotional state, teeth cleaning, and confidence to smile or speak. This result was similar to that found in the LGBTQIA+ population of a large city in the Brazilian Southeast region, which pointed to a higher prevalence of oral health impact on eating, teeth cleaning, and emotional state [19]. In turn, with the use of another instrument, the OHIP‐14, another study identified that psychological discomfort and inability, and physical pain were the domains most affected by oral health in this population [10].

The quality of life related to oral health presented an association with its self‐perception, which was similar to that found in research with this population group in the same country [10]. However, even in the group with a positive self‐perception of oral health, 44% of the participants in the present study presented some impact of oral health on daily activities.

In this study, the bivariate association between OIDP and income was not maintained in the final statistical model, which was observed in a study previously conducted with another oral health‐related quality of life instrument [10]. The self‐perception in oral health seems to be associated with income [36], which was also observed in the present study, between its lowest stratum and the fair self‐perception of oral health.

## 5 Conclusion

The research indicated disparities in subjective oral health measures in the LGBTQIA+ population, based on sociodemographic characteristics, experiences in dental services, and factors related to their use and treatment needs, and points to unprecedented associations between discrimination and subjective oral health outcomes in the adult Brazilian LGBTQIA+ population, in addition to reinforcing the greater vulnerability of the transgender population to these outcomes. These results contribute to highlighting the importance of health equity and fighting discrimination against sexual and gender minorities.

## Conflicts of Interest

The authors declare no conflicts of interest.
